# Ovalbumin-Derived Peptides Activate Retinoic Acid Signalling Pathways and Induce Regulatory Responses Through Toll-Like Receptor Interactions

**DOI:** 10.3390/nu12030831

**Published:** 2020-03-20

**Authors:** Mónica Martínez-Blanco, Leticia Pérez-Rodríguez, Daniel Lozano-Ojalvo, Elena Molina, Rosina López-Fandiño

**Affiliations:** Instituto de Investigación en Ciencias de la Alimentación (CIAL, CSIC-UAM), Nicolás Cabrera 9, 28049 Madrid, Spain; m.martinez.blanco@csic.es (M.M.-B.); leticia.p.r@csic.es (L.P.-R.); daniel.lozano@csic.es (D.L.-O.); e.molina@csic.es (E.M.)

**Keywords:** dendritic cells, peptide immunotherapy, regulatory T cells, retinoic acid, toll like receptors

## Abstract

This study investigates the potential of a hydrolysate of ovalbumin with pepsin (OP) to preclude Th2-type immunity by the enhancement of tolerogenic dendritic cells (DCs) and regulatory T (Treg) cells. Through Toll-like receptor (TLR) stimulation, OP enhances the retinoic acid pathway on DCs by means of the induction of aldehyde dehydrogenase enzymes and transforming growth factor beta (TGF-β), and it confers upon DC the ability to upregulate interleukin 10 (IL-10) as well as other tolerance-promoting mediators downstream of TRL signalling, such as IL-27, IL-33, Notch ligands, OX40L, and the transcription factors IRF4 and IRF8. OP-conditioned DCs induce the expansion of Foxp3+ and Tr1 cells in co-culture with CD4+ T cells. Furthermore, OP directly conditions CD4+ T cells from naïve mice, without the mediation of DCs, to express aldehyde dehydrogenase (ALDH) enzymes and, in the presence of the Th2 cytokine IL-4 and exogenous TGF-β, it enhances Foxp3 expression. It is noteworthy that, on CD4+ T cells isolated from egg-allergic mice, OP significantly enriches the levels of Foxp3+ and Foxp3+ RORγt+ CD4+ T cells. In conclusion, we show that food peptides may work, analogously to microbial-driven signals, through TLRs, to promote a tolerogenic phenotype on cells of the innate and adaptive immune system, a property that is further enhanced in the context of a Th2 cytokine-rich environment.

## 1. Introduction

Peripherally induced regulatory T (Treg) cells are considered essential for restraining immune responses directed towards innocuous food antigens in the intestinal mucosa [1]. Dendritic cells (DCs) drive the conversion of naïve T cells into Foxp3+ Treg cells by antigen presentation in the context of tolerogenic signals, depending on the microenvironmental milieu in which they are located. In this respect, the vitamin A metabolite retinoic acid (RA) deeply influences DC function, as it confers upon DCs migratory properties and promotes the secretion of TGF-β and IL-6 [2]. RA is provided by intestinal epithelial cells, macrophages, and stromal cells [3]. Moreover, DCs from the intestinal tract and associated lymphoid tissues express aldehyde dehydrogenase (ALDH) enzymes, in particular the ALDH1A2 isoform, which enable them to synthesise RA under the stimulus of RA itself, granulocyte-macrophage colony-stimulating factor (GM-CSF), IL-4, IL-13, and Toll-like receptor (TLR) activation [4,5,6]. RA exerts multiple roles in adaptive mucosal immunity: it is essential for the generation of IgA-secreting B cells [7], it enhances the expression of α4β7 gut homing integrin on T cells, and cooperates with TGF-β in the generation of Foxp3+ Treg cells [8,9], although it downregulates the anti-inflammatory cytokine IL-10 [10]. Furthermore, a RA-rich microenvironment can directly inhibit cytokine production by effector Th2 cells, contributing to impair allergic diseases while releasing the antagonist effect of Th2 cytokines on Treg cell generation [11].

Immunotherapy with hydrolysates of ovalbumin (OVA) and egg white (EW) with pepsin was shown to stimulate tolerance development in BALB/c mice allergic to EW more effectively than treatment with the intact allergens, by virtue of the induction of Treg cells [12,13]. Hydrolysate-treated mice exhibit upregulated intestinal expression of *Csf2*, *Aldh1a1*, *Aldh1a2*, and *Tgfb1*, that are involved in the conversion of vitamin A into RA. Furthermore, tolerance parallels the development of Foxp3+ CD4+ T cells that simultaneously express RORγt, and it has been shown that RA is a characteristic component of local milieus that preferentially drive the induction and expansion of functionally suppressive RORγt+ Treg cells [14,15]. Interestingly, a Th2-skewed environment, such as that type of food allergy, was favourable to the promotion of vitamin A metabolism and to the induction of a Foxp3+ RORγt+ phenotype on CD4+ T cells by the hydrolysates [13].

This study shows that OVA-derived peptides confer upon DCs the ability to upregulate RA and IL-10 through TLR stimulation. Peptide-conditioned bone marrow (BM)-DCs acquire a tolerogenic function, as they induce the expression of Foxp3 and IL-10 in CD4+ T cells. Furthermore, the hydrolysate of OVA directly conditions CD4+ T cells from naïve mice, without the mediation of DCs, to express ALDH enzymes and, in the presence of IL-4 and exogenous TGF-β, it enhances Foxp3 expression on these cells. It is noteworthy that, on CD4+ T cells from allergic mice, expression of the transcription factors Foxp3 and RORγt is further enhanced. Together, these results evidence that peptide immunotherapy, acting on cells of the innate and adaptive immune system, promotes the development of Treg cell subsets with different phenotypes.

## 2. Materials and Methods

### 2.1. Proteins, Hydrolysates, and Animals

OVA grade VI and porcine pepsin (EC 3.4.23.1, 3440 U mg^−1^) were purchased from Sigma-Aldrich (St. Louis, MO, USA). EW was obtained from fresh shell eggs and whey protein (WP, Lacprodan DE-9224K) was from Arla Foods (Sønderhøj, Denmark). The protein content of OVA, EW, and WP was determined by the Kjeldahl method. The lipopolysaccharide (LPS) level of proteins (substrates and enzyme) was quantified by the NF-κB assay on THP1- Blue reporter cells (Invivogen, Toulouse, France) [16] and, when necessary (just in the case of OVA), purification to less than 1 EU mg^−1^ was achieved with polymyxin columns (Thermo Scientific, Waltham, MA, USA).

OVA, EW, and WP were hydrolysed with pepsin (172 U/mg) at pH 1.5 and 37 °C for 24 h. The enzyme was inactivated by neutralisation to pH 7.0 and the hydrolysates (termed, respectively, OP, EP, and WPP) were centrifuged at 5000 x g, 4 °C for 10 min. The peptide composition of the hydrolysates, as estimated by reverse phase high performance liquid chromatography tandem mass spectrometry RP-HPLC-MS/MS, was previously reported [17,18]. Absence of contamination of the hydrolysates with LPS was confirmed and the possibility that the inactivated pepsin preparation could exert any immuno-stimulating effect was excluded using spleen cells from EW-sensitised mice [17]. The fraction with a molecular mass lower than 10 kDa, required for certain experiments, was obtained by ultrafiltration (Amicon Ultra, Millipore, Eschborn, Germany). Peptide sequences identified by RP-HPLC-MS/MS (ESI-MS/MS) in the fraction of OP with molecular mass lower than 10 kDa (OP < 10 kDa) are shown in Table S1. For cell culture assays, the concentration of the hydrolysates and their fractions was adjusted to 50 μg protein mL^−1^ according to their protein content, as determined by the bicinchoninic acid assay (BCA, Pierce BCA Protein Assay Kit, Thermo Scientific). This concentration was chosen after a dose-response assay on spleen cells from EW-sensitised mice [17].

Female BALB/c mice (6 weeks of age) were from Charles River Laboratories (Saint Germain Sur l’Arbresle, Rhône, France). For some experiments, in which an egg-allergic status was required, mice were sensitised by the intraperitoneal administration of 100 μg of EW plus 2.66 mg alum once a week over 3 weeks and sacrificed one week apart. Mice were verified for proper immunisation by analysis of serum-specific IgE and IgG1 antibodies [12].

All the animal experiments followed the European legislation (Directive 2010/63/UE) and were approved by Comunidad de Madrid (Ref PROEX 089/15).

### 2.2. Dendritic Cell Cultures

BM cells were isolated from femurs of naïve BALB/c mice and cultured for 10 days in RPMI-1640 medium supplemented with 10% Fetal Bovine Serum, 1% L-glutamine, 1% penicillin/streptomycin (Biowest SAS, Nuaillé, France), 100 mM sodium pyruvate, 50 µM β-mercaptoethanol, 10 mM HEPES and 10% non-essential amino acids (Sigma-Aldrich), containing 20 ng mL^−1^ GM-CSF (PeproTech, London, UK). DCs (CD11c^+^ cells) from mesenteric lymph nodes (MLN) and spleens were isolated using EasySep negative selection kits (StemCell Technologies, Vancouver, BC, Canada). DCs (1 × 10^6^ cells mL^−1^) were cultured for 24 h with medium alone (RPMI), proteins or hydrolysates (50 μg protein mL^−1^), or RA (100 nM or 1 μM; Sigma-Aldrich), in the absence or presence of 20 ng mL^−1^ IL-4 (PeproTech). In some experiments, DCs were incubated for 60 min with 10 μg mL^−1^ neutralising anti-TLR2 (TL2.1), anti-TLR4 (MTS510), or anti-TLR5 (Q23D11) antibodies, or isotype control (P3.6.2.8.1) (all from eBioscience, San Diego, CA, USA, except for anti-TLR5, which was from Invivogen). Before and after stimulation, DCs were analysed by flow cytometry. Following culture, cells were used for ALDH activity analyses or preserved for gene expression analyses.

### 2.3. Co-Cultures of Dendritic Cells and T Cells

Spleen CD4^+^ T cells were isolated using EasySep negative selection kits (StemCell Technologies). BM-DCs pulsed with different stimuli for 24 h, as above (1 × 10^5^ cells mL^−1^), were incubated with CD4^+^ T cells from naïve mice (1 × 10^6^ cells mL^−1^) in RPMI. After 2 days, cells were re-stimulated with 3 µg mL^−1^ anti-CD3 (17A2, eBioscience) and the co-culture was maintained for 2 additional days. Before and after co-culture, T cells were analysed by flow cytometry.

### 2.4. T Cell Cultures

CD4^+^ T cells from naïve or EW-sensitised mice (1 × 10^6^ cells mL^−1^) were cultured with medium alone (RPMI), proteins or hydrolysates (50 μg protein mL^−1^), RA (1 μM), or LPS (100 ng mL^−1^; Sigma-Aldrich) in the absence or presence of 5 ng mL^−1^ TGF-β (eBioscience). In some experiments, the ALDH inhibitor diethylaminobenzaldehyde (DEAB; Stemcell Technologies) was also added at a final concentration of 100 μM. After 2 days, cells were re-stimulated with 3 µg mL^−1^ anti-CD3 and anti-CD28 (37.51, eBioscience), and the culture was maintained for 2 additional days. Before and after stimulation, T cells were analysed by flow cytometry. Following culture, cells were used for ALDH activity analyses or preserved for gene expression analyses.

### 2.5. Gene Expression Analyses

RNA extraction and qPCRassays were performed as described [13]. Relative gene expression was calculated by the 2^−ΔΔCT^ method [19], normalising data to the expression of the *Actb* gene (coding for β-actin).

### 2.6. Flow Cytometry Analyses

Samples were stained with the following antibodies: anti-CD16/CD32 (93), anti-CD11c-PE-Cy7 (N418), anti-CD64-APC (X54-5/7.1), anti-CD103-PE (2E7), anti-MHCII-FITC (M5/114.15.2), anti-CD4-Alexa Fluor 700 (GK1.5), anti-IL-10-FITC (JES5-16E3), anti-GATA3-PerCP/Cy5.5 (16E10A23), anti-Foxp3-PE (150D/E4), and anti-RORγt-APC (B2D) (all from eBioscience, except for anti-GATA3-PerCP/Cy5.5, which was from Biolegend, San Diego, CA, USA), and live cells were determined with the LIVE/DEAD^®^ Fixable Near-IR Dead Cell Stain Kit (Thermo Fisher Scientific). ALDH activity was determined by using the ALDEFLUOR staining kit, following the manufacturer’s recommendations (Stemcell Technologies Inc., Vancouver, BC, Canada). ALDH-bright cells were detected in the fluorescein isothiocyanate (FITC) channel. Approximately 10^5^ cells were acquired with a Gallios flow cytometer and analyses were performed with Kaluza Analysis software (version 1.3) (Beckman Coulter, Krefeld, Germany).

### 2.7. Statistical Analyses

Results are presented as means ± SEMof 3 technical replicates of experiments representative of, at least, 3 biological replicates. Differences between a control and an experimental group were assessed by the unpaired two-tailed Student’s t-test and differences among three or more groups were determined by one-way analysis of variance (ANOVA), followed by Tukey’s post-hoc test, except for gene expression data, which were evaluated by the Mann–Whitney U test. *p* < 0.05 was considered statistically significant. Statistical analyses were performed using GraphPad Prism v5 (GraphPad Software Inc., San Diego, CA, USA).

## 3. Results

### 3.1. Dendritic Cells Pulsed with the Hydrolysate of Ovalbumin with PepsinAcquire Tolerogenic Properties

**Figure 1:**
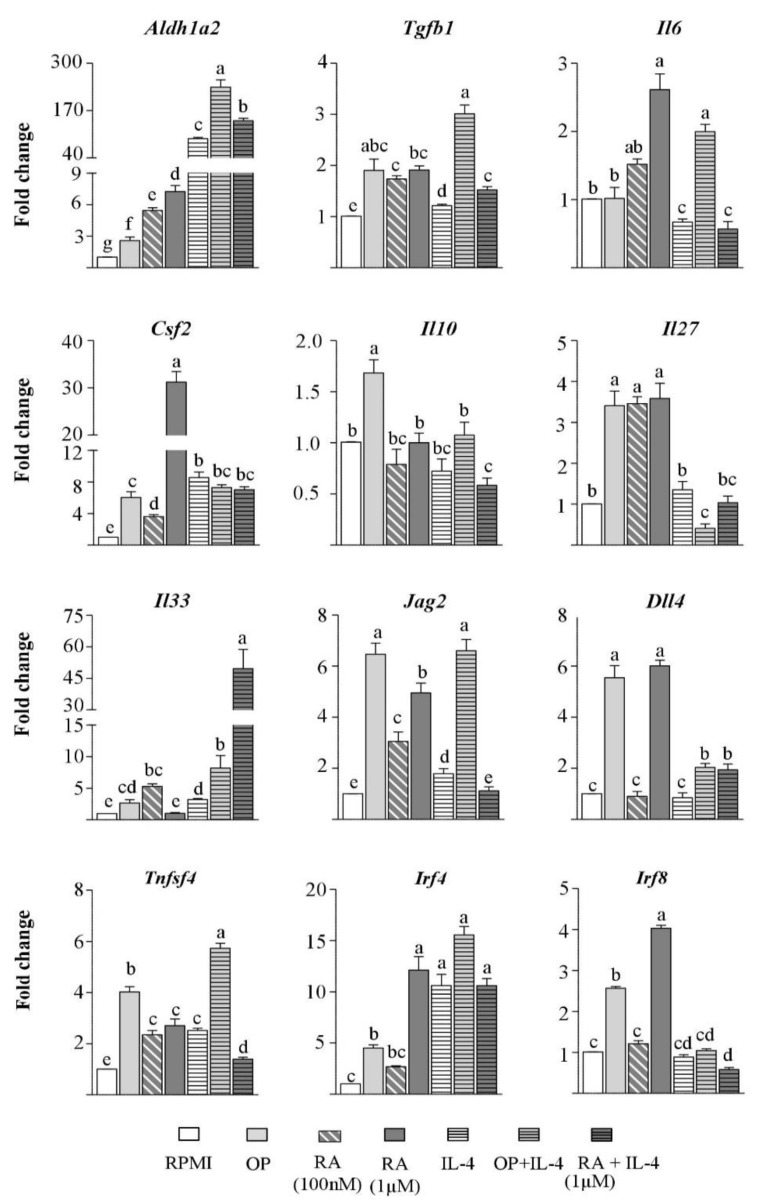
Bone marrow-dendritic cells (BM-DCs) pulsed with ovalbumin with pepsin (OP) acquire tolerogenic features. BM-DCs from naïve mice were cultured for 24 h without stimuli (RPMI), or with OP, retinoic acid (RA), IL-4, OP+IL-4, or RA+IL-4. Gene expression of *Aldh1a2*, *Tgfb1*, *Il6*, *Csf2, Il10*, *Il27, IL33*, *Jag2, Dll4*, *Tnfsf4, Irf4*, and *Irf8* was assayed by quantitative polymerase chain reaction (qPCR), normalised to the reference gene *Actb*, and expressed relative to BM-DCs cultured in RPMI. Results of an experiment representative of, at least 3, separate experiments performed in triplicate are shown. Data are means ± standard error of the mean (SEM). Different letters indicate statistically significant differences (*p* < 0.05) calculated using the Mann–Whitney U test.

The effect of OP on DC activation was studied. Incubation with the hydrolysate for 24 h enhanced the RA pathway on BM-DCs by upregulating Aldh1a2 and Tgfb1 expression, although that of *Il6* was not concomitantly increased (Figure 1). Furthermore, OP also induced the expression of *Csf2* (coding for GM-CSF) in the BM-DC culture (composed of, approximately, 90% CD11c+ CD64- cells and 10% CD11c+ CD64+ macrophages, not shown), and macrophage-produced GM-CSF is known to enhance ALDH1A2 activity in DCs [5]. These observations, together with the finding that BM-DCs stimulated with OP also overexpressed Il10 (Figure 1), suggest that they could be efficient tolerance inducers. We then looked at genes encoding additional mediators involved in the DC-driven promotion of regulatory responses, such as IL-27 [20], IL-33 [21], the Notch ligands Jagged2 and Delta4 [22], OX40L [23], and the interferon regulatory factors (IRF) IRF4 and IRF8, that specify DC development [24]. The results showed that OP increased the expression of *Il27*, *Il33*, *Jag2*, *Dll4* (coding for Jagged2 and Delta4, respectively), *Tnfsf4* (coding for OX40L), *Irf4*, and *Irf8* in BM-DCs (Figure 1).

The fraction of the hydrolysate with a molecular mass lower than 10 kDa exerted similar effects, although less pronounced regarding the expression of *Aldh1a2*, *Jag2*, and *Dll4*, which points toa contribution of high molecular mass peptides to the functionality of the hydrolysate (Figure S1). Additionally, we checked if the ability to promote a Treg cell-inducing gene signature in BM-DCs was common to other food protein hydrolysates, and found that EW and WP hydrolysed with pepsin (respectively EP and WPP) also promoted the expression of *Aldh1a2*, *Jag2*, and *Tnfsf4* (Figure S1).

There is evidence for a positive feedback loop of RA on its own synthesis by activation of ALDH enzymes or RA receptors, which induce DCs to produce RA, biologically active TGF-β, and IL-6 [4,6,25]. Accordingly, when used for BM-DC stimulation, RA, particularly at the highest concentration assayed (with the exception of *Il33*), enhanced the expression of *Aldh1a2*, *Tgfb1*, *Il6*, and *Gsf2,* as well as that of *Il27*, *Il33*, *Jag2*, *Dll4*, *Tnfsf4, Irf4*, and *Irf8* in BM-DCs (Figure 1). In general terms, RA and OP exerted analogous effects, except for their different influence on the regulation of *Il10* and *Il6* (Figure 1).

We then investigated the effect of OP on *Aldh1a2* expression in murine CD11c^+^ cells from intestinal (MLN) and extra-intestinal (spleen) lymphoid tissues. Unlike spleen-DCs, MLN-DCs, as well as GM-CSF-induced BM-DCs, considerably express *Aldh1a2* and produce RA [26,27]. OP and RA enhanced *Aldh1a2* expression in MLN-DCs, although the effect was more pronounced on spleen-DCs (Figure S2), an observation that could be explained by the lower basal expression of RA-synthesising enzymes in the latter [5,25]. On spleen-DCs, OP also significantly upregulated *Il10* and *Il27* expression (Figure S2B).

### 3.2. IL-4 Enhances the Effects of the Hydrolysate of Ovalbumin with Pepsin on Aldh1a2 Expression in Bone Marrow-Dendritic Cells

In view of the similarities found between the effects of OP and RA, and the earlier finding that IL-4 and IL-13 work together with RA to instruct DCs to acquire a mucosal phenotype and function [5,28,29], we tested the influence of IL-4 on the effects of OP. Activation of BM-DCs with IL-4 alone increased the expression of *Aldh1a2*, *Tgfb1*, *Csf2*, and the Th2-skewing factors [30,31]: *Il33*, *Jag2*, *Tnfsf4*, and *Irf4* (Figure 1), but, in agreement with previous findings, IL-4 did not enhance Il6 or Il10 expression [32]. As expected, IL-4 synergised with RA to increase *Aldh1a2* expression in BM-DCs [5,29], and it considerably enhanced *Il33* expression. The addition of IL-4 to OP also potentiated *Aldh1a2* expression (an effect corroborated in spleen-DCs, see supplementary material Figure S2B), and that of *Il33*, although it did not contribute to enhancing the expression of the other DC genes studied, except for *Il6*, *Tnfsf4*, and *Irf4* (Figure 1). It is noteworthy that the combination of OP or RA with IL-4 either diminished or abolished their stimulatory effect on the expression of the Th1 polarising factors: *Il27*, *Dll4*, and *Irf8* (Figure 1) [30,33].

### 3.3. The Hydrolysate of Ovalbumin with Pepsin does not Promote CD103 Expression on Bone Marrow-dendritic Cells

**Figure 2:**
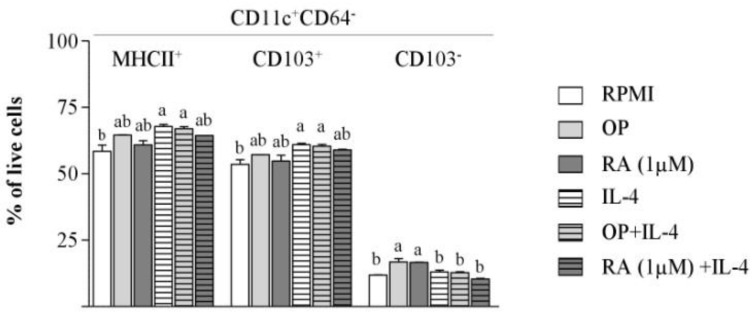
OP increases the proportion of CD11^+^ CD64^−^ CD103^−^ cells in BM-DC cultures. BM-DCs from naïve mice were cultured for 24 h without stimuli (RPMI), or with OP, RA, IL-4, OP+IL-4, or RA+IL-4. The percentage of MHCII^+^, CD103^+^, and CD103^−^ DCs (CD11c^+^ CD64^−^) cells within the total population of live cells was assayed by flow cytometry. Results of a representative experiment performed in triplicate are shown. Data are means ± SEM. Different letters indicate statistically significant differences (*p* < 0.05) calculated using one-way analysis of variance (ANOVA), followed by Tukey’s post-hoc test.

Culture of BM-DCs with OP enriched the proportion of CD11c+ CD64- cells and, in particular, the CD103- subset (Figure 2). Furthermore, RA treatment did not stimulate the expression of CD103 on CD11c+ CD64- cells either (Figure 2), in agreement with Feng et al. [26], who found that RA does not induce CD103 expression, despite the fact that it confers upon BM-DCs strong tolerogenic functions. Indeed, following the culture of BM-DCs with RA, there was an enhanced proportion of CD11c+ CD103- cells (Figure 2). Conversely, IL-4 and the combination of OP and IL-4 enhanced CD103, as well as MHCII expression, which points toa positive influence on DC maturation.

### 3.4. The Hydrolysate of Ovalbumin with Pepsin Enhances Aldh1a2 and Il10 Expression, and ALDH Activity in Dendritic Cells by Interacting with Toll-like Receptors

**Figure 3:**
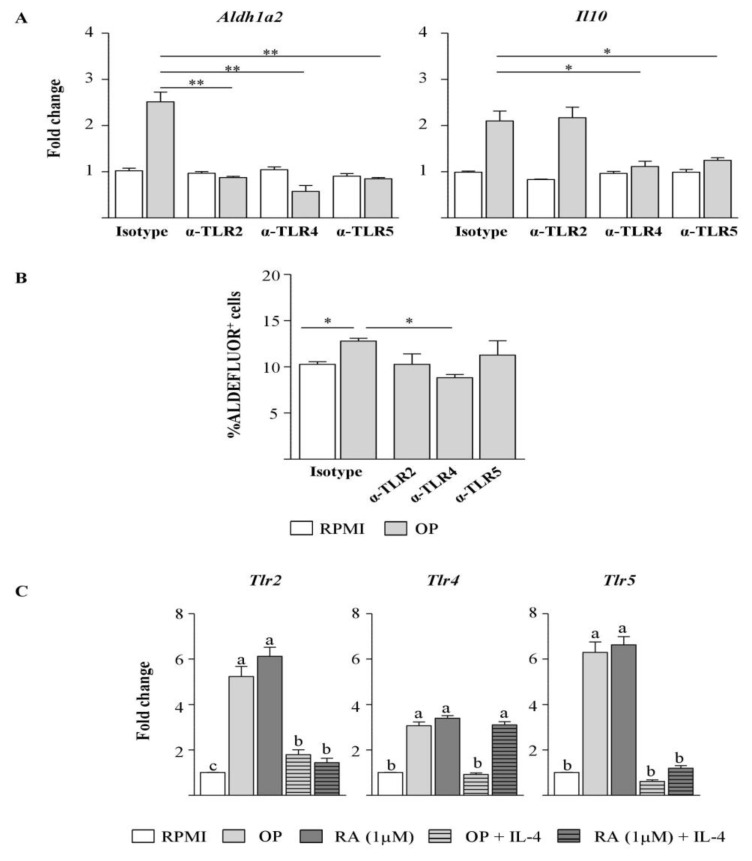
OP enhances *Aldh1a2* and *Il10* expression and aldehyde dehydrogenase (ALDH) activity in BM-DCs by interacting with TLRs. (**A**,**B**) BM-DCs from naïve mice were cultured for 24 h without stimuli (RPMI), or with OP in the presence of neutralising antibodies anti-TLR2, anti-TLR4, anti-TLR5, or isotype control. Gene expression of *Aldh1a2* and *Il10* was assayed by qPCR, normalised to the reference gene *Actb*, and expressed relative to BM-DCs cultured in RPMI with isotype control (**A**), and ALDH activity was assessed by the ALDEFLUOR assay (the ALDH inhibitor DEAB was used to determine baseline background fluorescence) (**B**). Results of an experiment representative of, at least, 3 separate experiments performed in triplicate are shown. Data are means ± SEM and statistically significant differences were calculated using unpaired two-tailed Student’s *t* test (* *p* < 0.05 and ** *p* < 0.01). (**C**) Gene expression of *Tlr2, Tlr4,* and *Tlr5* in BM-DCs cultured for 24 h without stimuli (RPMI) or with OP, RA, OP+IL-4, or RA+IL-4 was assayed by qPCR, normalised to the reference gene *Actb*, and expressed relative to BM-DCs cultured in RPMI. Data are means ± SEM (biological and technical triplicates). Different letters indicate statistically significant differences (*p* < 0.05) calculated using the Mann–Whitney U test.

Because it has been described that TLR signalling causes ALDH1A2 induction in DCs [6,26,27], we assessed the stimulation of BM-DCs with OP while inhibiting the pathways initiated by TLRs by the use of neutralising antibodies against the extracellular receptors TLR2, TLR4, and TLR5. Blockade of each of these TLRs abrogated the effect of OP on Aldh1a2 expression (Figure 3A) and decreased ALDH activity, as measured by the ALDEFLUOR assay, although only neutralisation of TLR4 led to a significant reduction (Figure 3B). Blockade of TLR4 and TLR5 also inhibited *Il10* expression (Figure 3A). Furthermore, the culture of BM-DCs with OP or RA enhanced the expression of *Tlr2*, *Tlr4*, and *Tlr5* (Figure 3C), an effect similarly observed on MLN-DCs (Figure S2A), while concomitant addition of IL-4 downregulated TLR genes in BM-DCs, with the only exception of RA plus IL-4 on *Tlr4* (Figure 3C).

### 3.5. The Hydrolysate of Ovalbumin with Pepsin Conditions Dendritic Cells to Induce a Regulatory Phenotype in CD4^+^ T cells, but also Promotes the Development of Regulatory T Cells without the Intermediation of Antigen Presenting Cells

**Figure 4:**
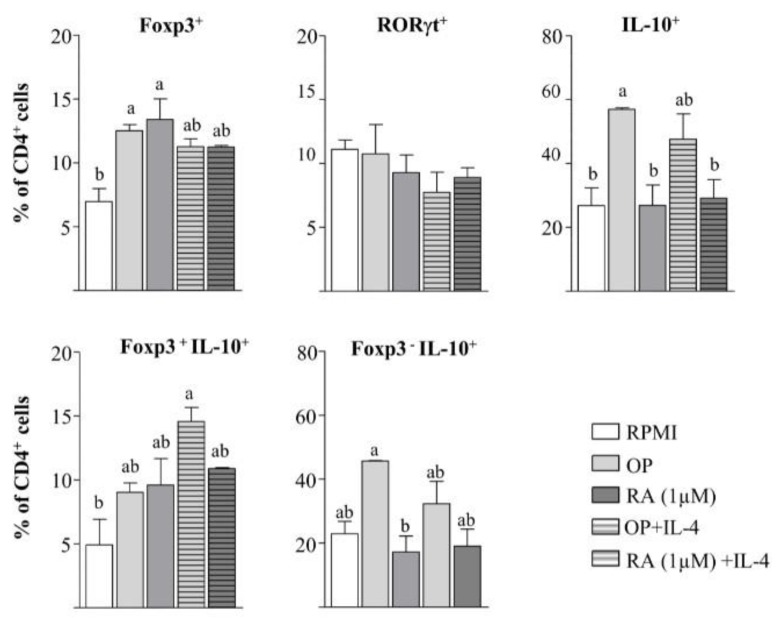
BM-DCs stimulated with OP enhance Foxp3^+^ Treg cells and Tr1 cells in co-culture with CD4^+^ T cells from naïve mice.BM-DCs, pre-conditioned for 24 h without stimuli (RPMI), or with OP, RA, IL-4, OP+IL-4, or RA+IL-4, were co-cultured for 2 days with spleen CD4^+^ T cells from naïve mice, followed by stimulation with anti-CD3 for 2 additional days. The percentage of Foxp3^+^, RORγt^+^, IL-10^+^, Foxp3^+^ IL-10^+^, and Foxp3^−^ IL-10^−^ cells within the total CD4^+^ T cell population was assayed by flow cytometry. Results of an experiment representative of 3 separate experiments performed in triplicate are shown. Data are means ± SEM. Different letters indicate statistically significant differences (*p* < 0.05) calculated using one-way ANOVA, followed by Tukey’s post-hoc test.

The effect of DCs incubated with different stimuli on CD4^+^ T cell phenotype was examined (Figure 4). OP- and RA-pulsed BM-DCs enhanced Foxp3 expression and did not change RORγt expression on co-cultured CD4^+^ T cells, although OP mainly increased the percentage of IL-10^+^ cells and, particularly, that of Foxp3^−^ IL-10^+^ cells (Tr1 cells). Despite the synergistic influence of the joint addition of OP or RA and IL-4 on *Aldh1a2* expression in BM-DCs, the level of Foxp3^+^ cells was not further enhanced with respect to OP or RA alone (Figure 4). This might be attributable to the observation that the combined treatments did not exert a significant added effect on *Tgfb1* expression in BM-DCs (Figure 1), and TGF-β, which was deliberately not added to the culture, is the limiting factor in Foxp3 differentiation [10].

**Figure 5:**
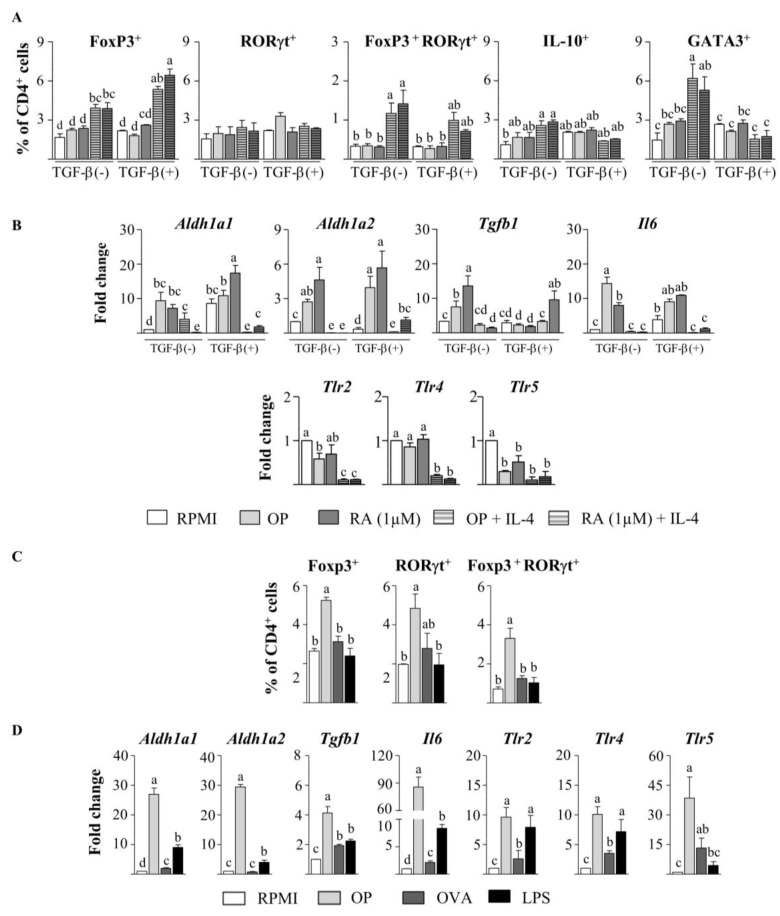
OP enhances Foxp3^+^ cells and Foxp3^+^ RORγ^+^ cells in culture with CD4^+^ T cells from EW-sensitised mice. (**A**,**B**) Spleen CD4^+^ T cells from naïve mice were cultured without stimuli (RPMI), or with OP, RA, OP+IL-4, or RA+IL-4 for 2 days, in the absence or presence of TGF-β, followed by stimulation with anti-CD3 and anti-CD28 for 2 additional days.The percentage of Foxp3^+^, RORγ^+^, Foxp3^+^ RORγ^+^, IL-10^+^, and GATA3^+^ cellswithin the total CD4^+^ T cell population was assayed by flow cytometry (**A**), and gene expression of *Aldh1a1, Aldh1a2, Tgfb1, Il6, Tlr2, Tlr4*, and *Tlr5* was assayed by qPCR, normalised to the reference gene *Actb*, and expressed relative to CD4^+^ T cells cultured in RPMI (**B**). (**C**,**D**) Spleen CD4^+^ T cells from EW-sensitised mice were cultured without stimuli (RPMI) or with OP, OVA, or LPS for 2 days, followed by stimulation with anti-CD3 and anti-CD28 for 2 additional days.The percentage of Foxp3^+^, RORγ^+^, and Foxp3^+^ RORγ^+^ cellswithin the total CD4^+^ T cell population was assayed by flow cytometry(**C**), and gene expression of *Aldh1a1, Aldh1a2, Tgfb1, Il6, Tlr2, Tlr4,* and *Tlr5* was assayed by qPCR, normalised to the reference gene *Actb*, and expressed relative to CD4^+^ T cells cultured in RPMI (**D**). Results of an experiment representative of 3 separate experiments performed in triplicate are shown. Data are means ± SEM. Different letters indicate statistically significant differences (*p* < 0.05) calculated using one-way ANOVA, followed by Tukey’s post-hoc test (**A**,**C**) or the Mann–Whitney U test (**B**,**D**).

Since T cells are known to express TLRs, and thus they could react to OP, we next assessed the effects of OP on CD4+ T cells stimulated with anti-CD3 and anti-CD28 without the intermediation of DCs. In the absence of exogenous TGF-β, neither OP nor RA increased the expression of Foxp3, RORγt, or IL-10 in CD4+ T cells from naïve mice (Figure 5A), despite the fact that they did upregulate *Aldh1a1*, *Aldh1a2*, *Tgfb1*, and *Il6* expression (Figure 5B) and increased ALDH activity, as measured by the ALDEFLUOR assay (Figure S3A). On the other hand, the expression of *Tlr2*, *Tlr4*, or *Tlr5* was not enhanced (Figure 5B). Similar results were obtained when TGF-β was added to the culture (Figure 5A,B). However, when combined with IL-4, OP and RA significantly enriched the proportion of Foxp3+ cells and double-positive Foxp3+ RORγt+ cells, despite the proportion of RORγt+ cells remaining unchanged (Figure 5C). OP+IL-4 and RA+IL-4 also induced Th2 differentiation, as they significantly augmented IL-10+ and GATA3+ cells. Notably, in the presence of TGF-β, the generation of Foxp3+ cells was promoted, while that of Th2 cells was inhibited (Figure 5A). After 96 h of the culture of CD4+ T cells from naïve mice with OP+IL-4 or RA+IL-4, no upregulation of *Aldh1a1*, *Aldh1a2*, *Tgfb1*, or *Il6* genes could be detected (Figure 5B). However, stimulation of CD4+ T cells in the presence of the ALDH inhibitor DEABovercame the enhancing effect of OP+IL-4 and RA+IL-4 on Foxp3 induction ( Figure S3B), thus showing the mediation of RA synthesis in Foxp3+ cell development.

To further assess the influence of a Th2-dominated environment, we used CD4+ T cells isolated from the spleen of mice intraperitoneally sensitised to EW to mimic an egg-allergic status (Figure 5C,D). In this setting, stimulation with OP, unlike stimulation with the intact OVA allergen, significantly increased the level of Foxp3+ cells, RORγt+ cells, and Foxp3+ RORγt+ cells (Figure 5C). Furthermore, OP strongly upregulated the expression of *Aldh1a1*, *Aldh1a2*, *Tgfb1*, *Il6*, *Tlr2*, *Tlr4*, and *Tlr5* in CD4+ T cells from sensitised mice, although no expression of Il10 was detected (not shown). Of note, the effect of OP on the genes that code for ALDH1A1, ALDH1A2, IL-6, and TGF-β was substantially higher than that exerted by the TLR2- and TLR4-agonist LPS (Figure 5D), which, in turn, failed to enhance the proportion of Foxp3+ cells (Figure 5C). Remarkably, sensitised mice were found to have higher MLN and spleen expression of *Tlr2*, *Tlr4*, and *Tlr5* than naïve mice (Figure S4). Therefore, under the influence of an allergic status, OP favoured RA metabolism and the expansion of the highly suppressive RORγt+ Treg subset [14,15], acting directly on CD4+ T cells.

## 4. Discussion

Intestinal DCs, with the help of other cells involved in innate immunity, such as macrophages and nearby intestinal epithelial cells, are known to play a crucial role in the generation of regulatory adaptive immune responses that avoid food allergy. Accordingly, and in view of the enhanced therapeutic effect of hydrolysed egg proteins compared to their intact counterparts [12], this study investigates the potential of peptides to preclude Th2-type immunity by the enhancement of tolerogenic DCs and effector Treg cells.

BM-DCs, in addition to intestinal (MLN) and extra-intestinal (spleen) DCs, upregulated *Aldh1a2* in response to OP. In mice, GM-CSF-differentiated BM-DCs, MLN- and spleen-DCs express most TLRs [34,35], whose activation contributes to RA production, even in spleen-DCs which are deficient in ALDH enzymes [6,25,26,27,35]. The observation that blocking antibodies specific for TLR2, TLR4, and TLR5 inhibited the effects of OP on *Aldh1a2* expression in BM-DCs indicates that dietary peptides confer upon DCs the property to synthesise RA through TLR stimulation, also suggesting that the ability to sense the hydrolysate with a combination of several receptors may facilitate tolerogenic responses in cells of the innate immune system. Our results, which are in line with studies showing activation of several TLRs in TLR-reporter cell lines by food proteins and their hydrolysates [36], suggest that the capacity to regulate the expression of genes involved in RA metabolism in DCs depend on peptide size and sequence Table S1. It should be noted that incubation of DCs with OP or with RA significantly augmented *Tlr2, Tlr4,* and *Tlr5* mRNA, which would help to reinforce TLR-mediated effects. Accordingly, expression of TLRs was previously shown to be directly and indirectly (through IL-27) upregulated by TLR stimulation, as well as by RA [6,37,38].

The effects of OP on BM-DCs were similar to those exerted by RA. In fact, regulation of DCs through RA requires MyD88, an adaptor protein which is conventionally associated with TLR signalling and mediates some of the microbiota’s tolerance-inducing pathways in the gastrointestinal tract [6,35,39]. However, unlike RA [4], OP significantly upregulated *Il10* and downregulated *Il6*, possibly because TLR signalling on DCs enhances IL-10 independently of the induction of ALDH1A2 activity with IL-10, together with RA, acting in an autocrine manner to suppress the production of pro-inflammatory cytokines, such as IL-6, via induction of the negative regulator suppressor of cytokine signalling 3 (SOCS3) [25].

Additional potential connections between the triggering of TLRs and the promotion of tolerogenic features on DC may lay in the induction of IL-27, IL-33, Notch ligands from the Jagged and Delta family, and the transcription factors IFR4 and IFR8, whose expression is activated downstream of the MyD88-dependent pathway [20,24,40,41]. Indeed, the effects of both OP and RA could be traced to the upregulation of *Il27*, *Il33, Jag2, Dll4*, *Tnfsf4, Irf4*, and *Irf8* in BM-DCs. IL-27 supports IL-10 production by CD4^+^ and CD8^+^ T cells [20], IL-33 promotes RA signalling in CD4^+^ T cells and stimulates Treg cell responses [21,42], while binding of Notch ligands enhances Foxp3 expression in CD4^+^ T cells and helps to maintain Treg cells in vitro and in vivo [22]. In particular, the Notch family ligands Jagged 1 and 2 have been related with the promotion of TGF-β signalling and Foxp3 transcription, while concomitant OX40L–OX40 interaction delivers survival signs, allowing Treg cell expansion [23].

On the other hand, IRF4 and IRF8 stimulate DC development and influence T cell skewing [24]. In this respect, whereas mucosal IRF4-dependent CD103^+^ CD11b^+^ DCs have been regarded as the main carriers of RA-converting enzymes [43], other studies have made it apparent that IRF8-dependent CD103^+^ CD11b^−^ DCs also display ALDH activity, which enables them to act as Treg cell-inducer subset [44,45]. Furthermore, expression of CD103 was found not to be required for DC to exert tolerogenic functions [6,35,46]. Indeed, stimulation of BM-DCs with OP and RA simultaneously increased *Aldh1a2* expression and the population of CD11c^+^ CD65^−^ CD103^−^ cells, reinforcing the concept that the environment confers upon DCs of different phenotypes the functional property to differentiate naïve T cells into Treg cells.

OP and RA-conditioned BM-DCs exerted tolerogenic actions, as they enhanced the expression of Foxp3 on co-cultured CD4^+^ T cells. Besides, OP distinctively stimulated the expression of IL-10 in Foxp3^−^ cells. Indeed, the activation of DCs with certain TLR ligands has been reported to induce potent immunosuppressive Foxp3^−^ IL-10^+^ cells (Tr1 cells) through the production of IL-10, but mainly of IL-27, with TGF-β further increasing IL-10 production by T cells [47]. Conversely, RA, despite promoting *Il27* expression, did not favour the development of IL-10-competent cells or Tr1 cells. Whereas RA does not impair IL-10 induction in mature Treg cells, it potently inhibits TGF-β-mediated induction of IL-10 in developing Foxp3^+^ cells through repression of IL-10 transcription, likely downstream of STAT-3-dependent signalling activated by IL10 and IL-27 [10]. The reciprocal effects of IL-27 and RA, produced by OP-pulsed DCs, on the TGF-β-mediated induction of IL-10 and Foxp3 in naive Treg cells, may allow the differentiation of different proportions of Tr1 and Foxp3^+^ cells depending on the molecular context, which would favour regulatory functions under certain circumstances when Foxp3 expression is compromised, such as inflammatory conditions. Interestingly, Dawicki et al. [48] found a novel phenotype of CD4^+^ Foxp3^−^ IL-10^−^ T cells, effective towards Th2 responses in food allergy, which are induced by DC differentiated in the presence of RA and exposed to TLR stimulation (with LPS), showing that the modulation of RA and IL-27 levels by TLR ligands modifies the balance of Treg cell subsets with different properties.

It is known that TLRs expressed on T cells may directly control immune responses in the absence of antigen-presenting cells. Thus, commensal bacteria mediate the conversion of CD4^+^ T cells into Foxp3^+^ cells via TLR2 signalling [49], and MyD88-dependent microbial sensing by Treg cells themselves favours humoral and cellular immunity [50]. Flagellin, a TLR5 agonist, potently enhances the expression of Foxp3 and the suppressive capacity of CD4^+^ CD25^+^ Treg cells [51], and similar results have been reported for agonists of TLR2 and TLR4 [52,53]. T cell receptor stimulation of CD4^+^ T cells from naïve mice in the presence of OP, without DCs, upregulated *Aldh1a1, Aldh1a2, Tgfb1,* and *Il6* expression and exhibited ALDH activity, although the proportion of Foxp3^+^ Treg and Tr1 cells were not concomitantly increased. Although information regarding ALDH expression and subsequent production of RA by CD4^+^ T cells is very scarce, Kanakry et al. [54] revealed that allogenic, and likely other types of stimulation, increase ALDH activity in conventional CD4^+^ T cells, in particular in Treg cells, allowing resistance to cytotoxic agents and immunological tolerance.

We hypothesised that a Th2 cytokine environment could cooperate with the hydrolysate in the induction of a tolerogenic phenotype in DCs, since as previously reported, IL-4 increases *Aldh1a2* expression in DCs and it acts synergistically with GM-CSF, RA, and TLR ligands [5,29]. In fact, DCs treated with RA plus IL-4 or with IL-4 alone enhance Foxp3^+^ cell conversion in the presence of TGF-β [5,29]. The simultaneous addition of OP and IL-4 upregulated the expression of *Aldh1a2, Il6,* and *Il33,* and downregulated *Il10* and *Il27* in BM-DCs, although these DCs did not exhibit a superior ability to induce Foxp3^+^ T cells, at least when exogenous TGF-β was not added to the co-culture. However, in the absence of antigen-presenting cells, the combinations of RA and OP with IL-4 drove the differentiation of CD4^+^ cells from naïve mice into IL-10^+^ and GATA3^+^ T cells, but also into Foxp3^+^ and Foxp3^+^ RORγt^+^ T cells, with the addition of TGF-β further inhibiting GATA3^+^ T cells and enhancing Foxp3 induction. This shows that OP facilitated the expansion of Treg cells over Th2 cells in the presence of the Th2 hallmark cytokine IL-4. IL-4 has been reported to have stimulatory and inhibitory effects on Treg cell development and maintenance [55]. Indeed, while IL-4 may block the generation of TGF-β-induced Foxp3^+^ Treg cells [56], RA releases IL-4-mediated repression of Foxp3 differentiation [57]. Interestingly, a recent report revealed that RA transforms human group 2 innate lymphoid cells into regulatory innate lymphoid cells and that this conversion is further enhanced during Th2 inflammation, working as a negative feedback system for maintaining homeostasis [56]. Our results, showing abrogation of Foxp3 induction by OP+IL-4 when RALDH activity was inhibited with DEAB, indicate that direct RA production by CD4^+^ T cells themselves favoured Treg generation. Of note, the availability of IL-6 in a Th2-dominated environment, that could arise from the combined action of TLR activation and IL-4 [32], would favour the generation of Treg cells bearing simultaneously the transcription factors Foxp3 and RORγt, since TGF-β plus IL-6 open the RORγt differentiation pathway and additional RA favours the generation of double-positive cells in vitro [14].

It is noteworthy that OP induced much higher levels of *Aldh1a1, Aldh1a2, Tgfb1,* and *Il6* expression in CD4^+^ T cells from EW-sensitised mice than in cells from naïve mice. Furthermore, basal expression of *Tlr2, Tlr4,* and *Tlr5* mRNA was greater in MLNs and spleens from sensitised mice as compared with naïve mice, suggesting a more pronounced susceptibility to TLR signalling. In fact, anti-CD3-induced activation is known to promote the expression of *Tlr2*, *Tlr4,* and *Tlr5* in CD4^+^ T cells from BALB/c mice, with antigen-experienced cells, such as effector or effector memory cells, displaying higher levels of expression than naïve cells [58]. Remarkably, on CD4^+^ T cells from EW-sensitised mice, OP significantly enhanced the levels of Foxp3^+^ and Foxp3^+^ RORγt^+^ CD4^+^ T cells, the stable regulatory subset positively associated with RA metabolism which abounds in the intestinal tissues of mice bearing a complex microbiota [15,59], further showing that exposure to a Th2 cytokine-rich environment helped OP to induce ALDH enzymes and promote a tolerogenic phenotype.

In conclusion, we show that peptides in hydrolysed proteins or digested foods may work, analogously to microbial-driven signals, through TLRs to promote mucosal tolerance at different levels: TLR engagement on DCs indirectly enhanced Tr1 and Treg responses, while direct action on CD4^+^ T cells also mediated the expansion of Foxp3 cells and cells co-expressing Foxp3 and RORγt. Induction of ALDH activity in DCs and CD4^+^ T cells was central to these effects, with Th2-derived factors contributing to the regulatory action of peptides on cells of the innate and adaptive immune system.

## Supplementary Materials

The following are available online at https://www.mdpi.com/2072-6643/12/3/831/s1, Figure S1: Gene expression inBM-DCs from naïve mice cultured for 24 h with different stimuli; Figure S2: Gene expression inMLN-DCs (A) and spleen-DCs (B) from naïve mice cultured for 24 h with different stimuli; Figure S3: ALDH activityin spleen CD4^+^ T cells from naïve mice cultured with different stimuli; Figure S4: Gene expression in MLNs (A) and spleens (B) from naïve and EW-sensitized mice; Table S1: Peptide sequences identified by RP-HPLC-MS/MS (ESI-MS/MS) in the fraction of OP with molecular mass lower than 10 kDa (OP < 10 kDa).

## Abbreviations

ALDH: aldehyde dehydrogenase; BM, bone marrow; DC, dendritic cell; DEAB, diethylaminobenzaldehyde; EP, egg white hydrolysed with pepsin; EW, egg white; Foxp3, forkhead box P3; GM-CSF, granulocyte-macrophage colony-stimulating factor; Ig, immunoglobulin; IL, interleukin; IRF, interferon regulatory factor; LPS, lipopolysaccharide; MLN, mesenteric lymph node; OP, ovalbumin hydrolysed with pepsin; OVA, ovalbumin; qPCR, quantitative polymerase chain reaction; RA, retinoic acid; RALDH, retinaldehyde dehydrogenase; RORγt, RAR-related orphan nuclear receptor gamma t; SEM, standard error of the mean; TLR, Toll-like receptor; TGF-β, transforming growth factor beta; Treg cell, regulatory T cell; WP, whey protein; WPP, whey protein hydrolysed with pepsin.
