# Supplementary material for: Ovalbumin-Derived Peptides Activate Retinoic Acid Signalling Pathways and Induce Regulatory Responses Through Toll-Like Receptor Interactions
Source: Nutrients. 2020 Mar 20;12(3):831. doi: 10.3390/nu12030831 (PMC7146383; doi:10.3390/nu12030831)
Supplement: Supplementary file 1 [file nutrients-12-00831-s001.zip › Suppl Figure 1.pdf]

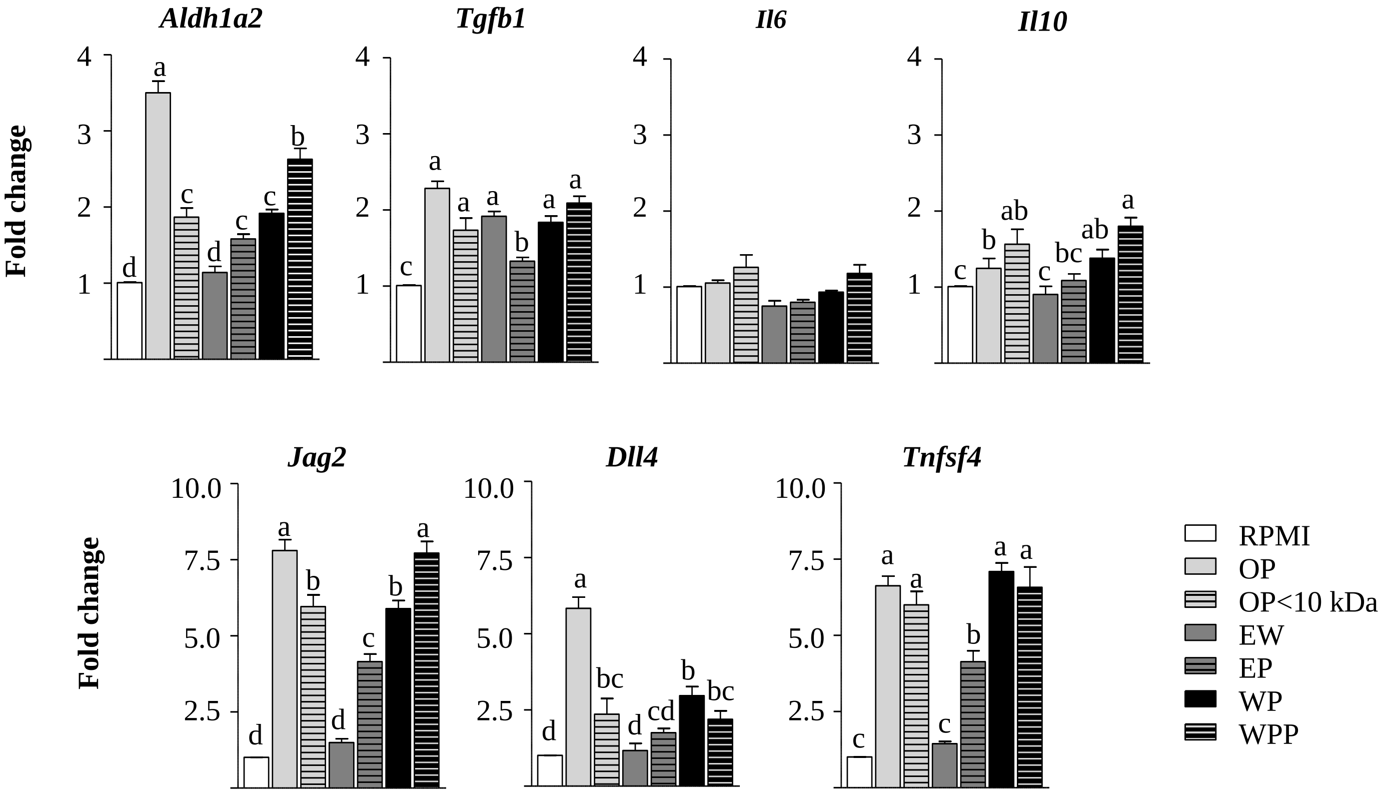

Supplemental Figure 1. Gene expression in BM-DCs from naïve mice cultured for 24 h with different stimuli [OP, the fraction of OP with molecular mass lower than 10 kDa (OP<10 kDa), EW, EW hydrolysed with pepsin (EP), WP, or WP hydrolysed with pepsin (WPP)], assayed by qPCR, normalized to the reference gene *Actb*, and expressed relative to BM-DCs cultured in RPMI. Data are means ± SEM (biological and technical triplicates). Different letters indicate statistically significant differences ($p < 0.05$) calculated using Mann-Whitney U test.
