# Supplementary material for: Ovalbumin-Derived Peptides Activate Retinoic Acid Signalling Pathways and Induce Regulatory Responses Through Toll-Like Receptor Interactions
Source: Nutrients. 2020 Mar 20;12(3):831. doi: 10.3390/nu12030831 (PMC7146383; doi:10.3390/nu12030831)
Supplement: Supplementary file 1 [file nutrients-12-00831-s001.zip › Suppl Figure 2.pdf]

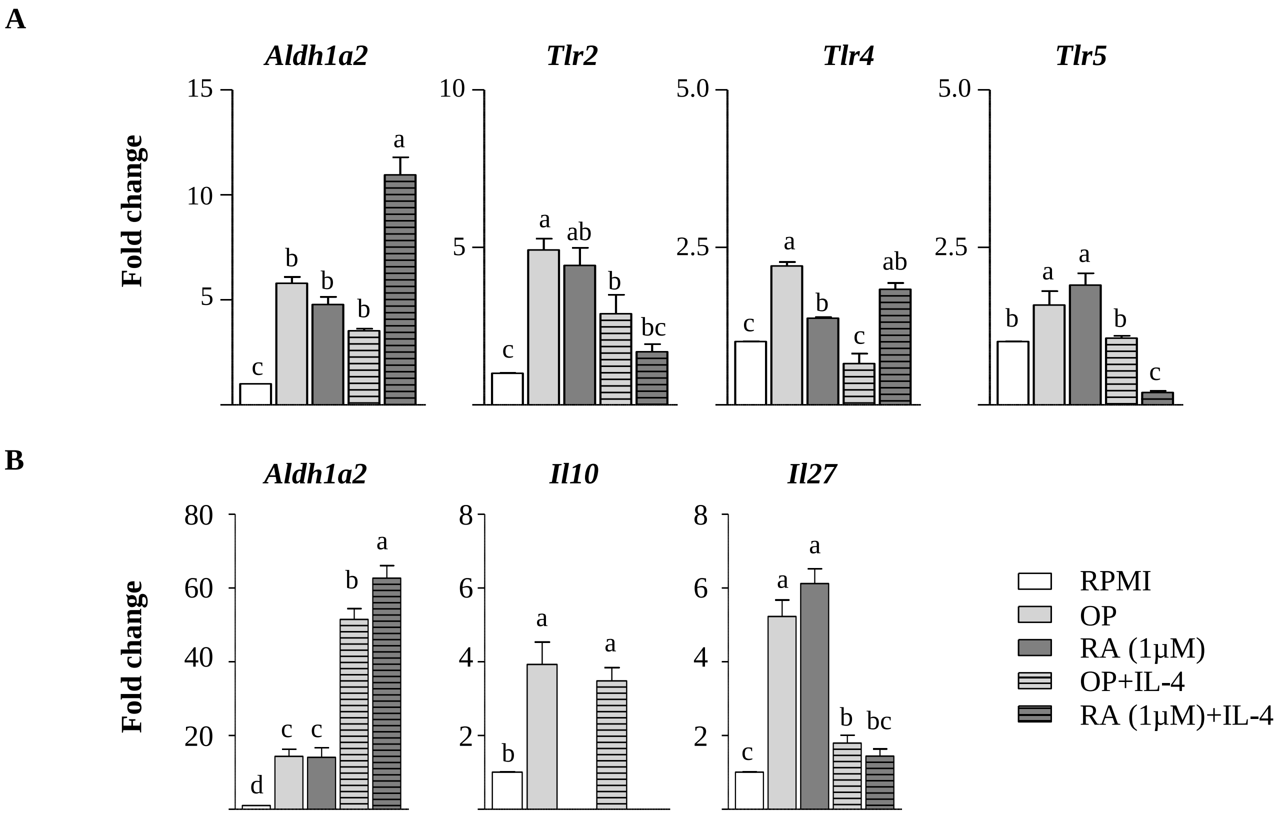

Supplemental Figure 2. Gene expression in MLN-DCs (**A**) and spleen-DCs (**B**) from naïve mice cultured for 24 h with different stimuli (OP, RA, OP+IL-4, or RA+IL-4), assayed by qPCR, normalized to the reference gene *Actb*, and expressed relative to DCs cultured in RPMI. Data are means ± SEM (biological and technical triplicates). Different letters indicate statistically significant differences ($p < 0.05$) calculated using Mann-Whitney U test.
