# Supplementary material for: Ovalbumin-Derived Peptides Activate Retinoic Acid Signalling Pathways and Induce Regulatory Responses Through Toll-Like Receptor Interactions
Source: Nutrients. 2020 Mar 20;12(3):831. doi: 10.3390/nu12030831 (PMC7146383; doi:10.3390/nu12030831)
Supplement: Supplementary file 1 [file nutrients-12-00831-s001.zip › Suppl Figure 3.pdf]

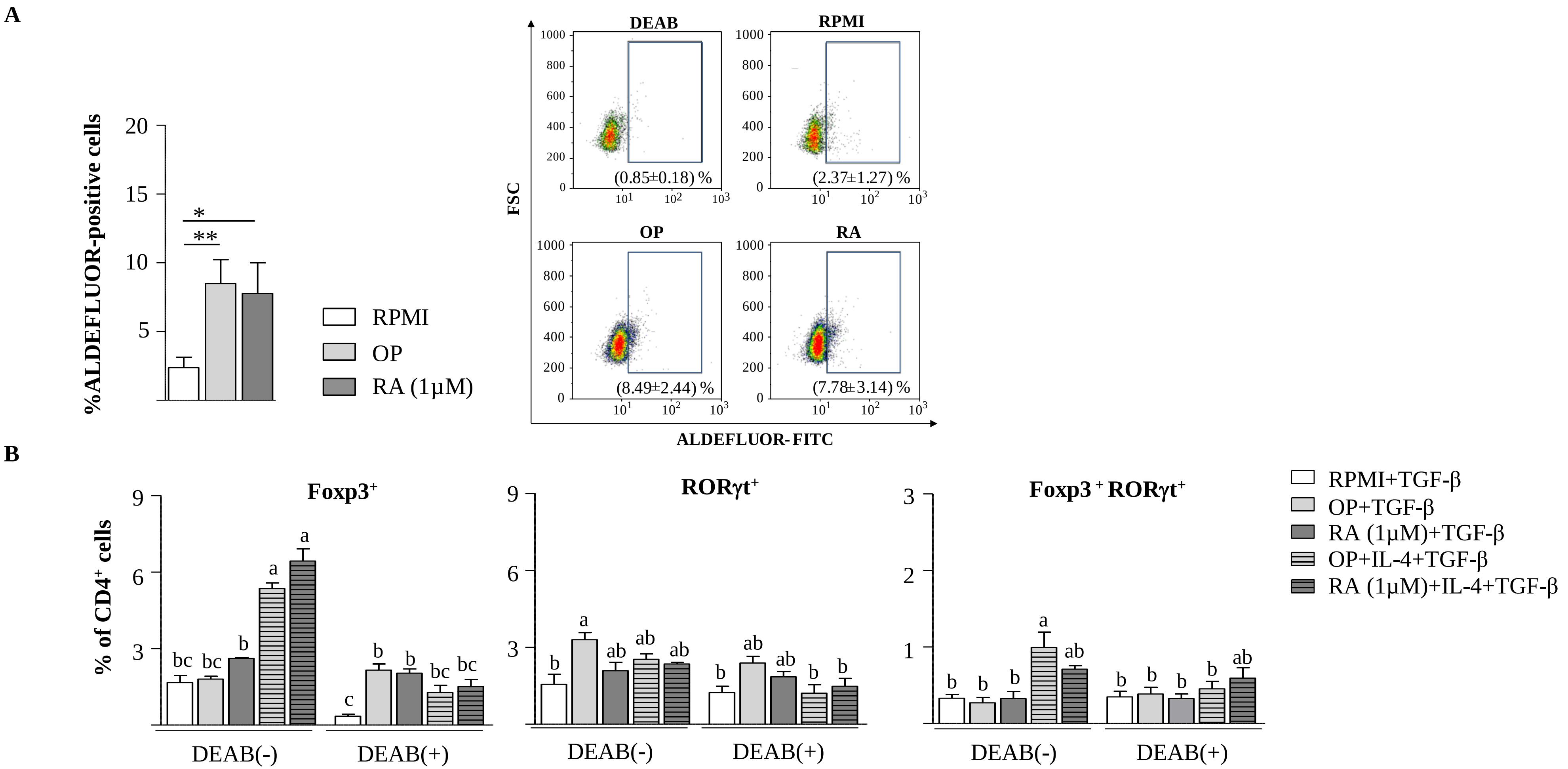

Supplemental Figure 3. A. ALDH activity, as assessed by ALDEFLUOR assay, in spleen CD4$^+$ T cells from naïve mice cultured without stimuli (RPMI), or with OP or RA for 2 days, followed by stimulation with anti-CD3 and anti-CD28 for 2 additional days. The ALDH inhibitor DEAB was used to determine baseline background fluorescence. Data are means ± SEM (biological and technical triplicates) and statistically significant differences were calculated using unpaired two-tailed Student's t test (* $p < 0.05$ and ** $p < 0.01$). **B.** Percentage of Foxp3$^+$, RORγ$^+$ and Foxp3$^+$ RORγ$^+$ cells within the total CD4$^+$ T cell population in spleen from naïve mice cultured with TGF-β without stimuli (RPMI), or with OP, RA, OP+IL-4, or RA+IL-4 for 2 days, in the absence or presence of the ALDH inhibitor DEAB, followed by stimulation with anti-CD3 and anti-CD28 for 2 additional days. Data are means ± SEM (biological and technical triplicates). Different letters indicate statistically significant differences ($p < 0.05$) calculated using one-way ANOVA, followed by Tukey post-hoc test.
