# Supplementary material for: Ovalbumin-Derived Peptides Activate Retinoic Acid Signalling Pathways and Induce Regulatory Responses Through Toll-Like Receptor Interactions
Source: Nutrients. 2020 Mar 20;12(3):831. doi: 10.3390/nu12030831 (PMC7146383; doi:10.3390/nu12030831)
Supplement: Supplementary file 1 [file nutrients-12-00831-s001.zip › Suppl Figure 4.pdf]

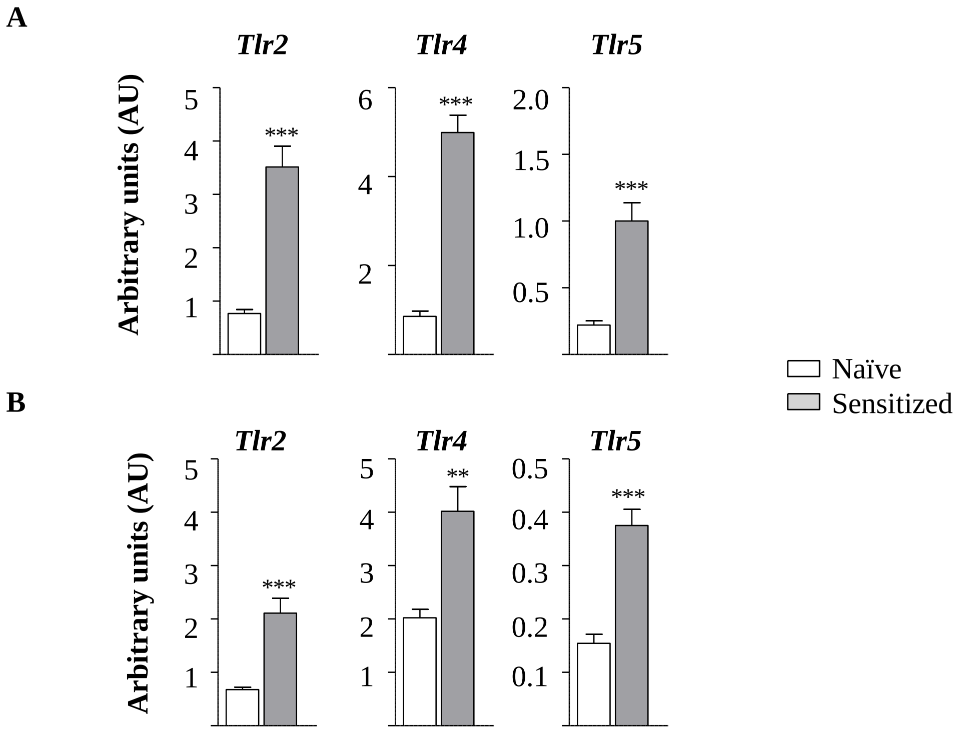

Supplemental Figure 4. Gene expression in MLNs (**A**) and spleens (**B**) from naïve and EW-sensitized mice, assayed by qPCR and normalized to the reference gene *Actb*. Data are means ± SEM (biological and technical triplicates). Different letters indicate statistically significant differences (** $p < 0.01$ and *** $p < 0.001$) calculated using Mann-Whitney U test.
