# Supplementary material for: Ovalbumin-Derived Peptides Activate Retinoic Acid Signalling Pathways and Induce Regulatory Responses Through Toll-Like Receptor Interactions
Source: Nutrients. 2020 Mar 20;12(3):831. doi: 10.3390/nu12030831 (PMC7146383; doi:10.3390/nu12030831)
Supplement: Supplementary file 1 [file nutrients-12-00831-s001.zip › Table S1.docx]

**Table S1.** Peptide sequences identified by RP-HPLC-MS/MS (ESI-MS/MS) in the fraction of OP with molecular mass lower than 10 kDa (OP<10 kDa).

| **Measured M/z** | **Calculated MH+** | **z** | **Mascot Score** | **Rt(min)** | **Range** | **Sequence** |
| --- | --- | --- | --- | --- | --- | --- |
| **1204.6** | 1204.6 | 1+ | 23 | 20.8 | 17 - 26 | KELKVHHANE |
| **947.5** | 947.5 | 1+ | 22 | 20.5 | 19 - 26 | LKVHHANE |
| **834.4** | 834.4 | 1+ | 16 | 16.0 | 20 - 26 | KVHHANE |
| **566.2** | 566.3 | 1+ | 13 | 31.5 | 30 - 34 | YCPIA |
| **522.2** | 522.3 | 1+ | 19 | 30.4 | 42 - 46 | VYLGA |
| **765.4** | 765.4 | 1+ | 8 | 26.5 | 42 - 48 | VYLGAKD |
| **953.5** | 953.5 | 1+ | 45 | 26.3 | 42 - 50 | VYLGAKDST |
| **1210.6** | 1210.6 | 1+ | 15 | 26.0 | 42 - 52 | VYLGAKDSTRT |
| **783.4** | 1565.8 | 2+ | 11 | 29.2 | 42 - 55 | VYLGAKDSTRTQIN |
| **948.5** | 948.5 | 1+ | 9 | 19.7 | 44 - 52 | LGAKDSTRT |
| **660.0** | 1318.7 | 2+ | 16 | 19.9 | 45 - 56 | GAKDSTRTQINK |
| **707.3** | 707.4 | 1+ | 8 | 13.9 | 47 - 52 | KDSTRT |
| **948.4** | 948.5 | 1+ | 12 | 23.4 | 47 - 54 | KDSTRTQI |
| **1062.6** | 1062.6 | 1+ | 7 | 20.2 | 47 - 55 | KDSTRTQIN |
| **676.4** | 676.4 | 1+ | 10 | 37.5 | 61 - 66 | DKLPGF |
| **848.4** | 848.4 | 1+ | 25 | 34.8 | 61 - 68 | DKLPGFGD |
| **566.1** | 566.2 | 1+ | 15 | 13.9 | 72 - 77 | AQCGTS |
| **665.3** | 665.3 | 1+ | 10 | 21.8 | 72 - 78 | AQCGTSV |
| **779.3** | 779.3 | 1+ | 6 | 19.0 | 72 - 79 | AQCGTSVN |
| **878.4** | 878.4 | 1+ | 7 | 27.3 | 72 - 80 | AQCGTSVNV |
| **755.4** | 755.4 | 1+ | 21 | 28.6 | 78 - 84 | VNVHSSL |
| **656.3** | 656.3 | 1+ | 21 | 25.0 | 79 - 84 | NVHSSL |
| **758.4** | 758.4 | 1+ | 6 | 28.3 | 85 - 90 | RDILNQ |
| **929.5** | 929.5 | 1+ | 13 | 20.9 | 89 - 96 | NQITKPND |
| **1028.5** | 1028.5 | 1+ | 12 | 25.6 | 89 - 97 | NQITKPNDV |
| **687.3** | 687.4 | 1+ | 17 | 19.7 | 91 - 96 | ITKPND |
| **949.5** | 949.5 | 1+ | 18 | 28.1 | 91 - 98 | ITKPNDVY |
| **1036.5** | 1036.5 | 1+ | 12 | 26.1 | 91 - 99 | ITKPNDVYS |
| **533.1** | 533.3 | 1+ | 16 | 22.8 | 101 - 105 | SLASR |
| **646.4** | 646.4 | 1+ | 5 | 32.9 | 101 - 106 | SLASRL |
| **667.2** | 667.3 | 1+ | 11 | 21.1 | 107 - 111 | YAEER |
| **830.4** | 830.4 | 1+ | 21 | 24.4 | 107 - 112 | YAEERY |
| **790.4** | 790.4 | 1+ | 19 | 38.9 | 110 - 115 | ERYPIL |
| **719.4** | 719.4 | 1+ | 20 | 24.7 | 119 - 124 | LQCVKE |
| **832.4** | 832.5 | 1+ | 43 | 34.8 | 119 - 125 | LQCVKEL |
| **807.4** | 807.4 | 1+ | 6 | 29.2 | 125 - 131 | LYRGGLE |
| **694.4** | 694.4 | 1+ | 7 | 24.8 | 126 - 131 | YRGGLE |
| **1018.5** | 1018.5 | 1+ | 5 | 32.0 | 126 - 134 | YRGGLEPIN |
| **1165.6** | 1165.6 | 1+ | 12 | 42.3 | 126 - 135 | YRGGLEPINF |
| **633.2** | 633.3 | 1+ | 18 | 13.8 | 136 - 141 | QTAADQ |
| **704.2** | 704.3 | 1+ | 10 | 16.8 | 136 - 142 | QTAADQA |
| **731.4** | 731.4 | 1+ | 6 | 26.7 | 143 - 148 | RELINS |
| **747.3** | 747.4 | 1+ | 9 | 29.8 | 146 - 151 | INSWVE |
| **749.3** | 749.3 | 1+ | 11 | 25.2 | 149 - 154 | WVESQT |
| **920.4** | 920.4 | 1+ | 5 | 24.3 | 149 - 156 | WVESQTNG |
| **734.3** | 734.3 | 1+ | 12 | 14.9 | 150 - 156 | VESQTNG |
| **506.0** | 506.2 | 1+ | 17 | 7.5 | 152 - 156 | SQTNG |
| **727.5** | 727.5 | 1+ | 17 | 36.0 | 157 - 162 | IIRNVL |
| **1126.6** | 1126.7 | 1+ | 9 | 33.6 | 157 - 166 | IIRNVLQPSS |
| **630.3** | 630.3 | 1+ | 28 | 21.5 | 161 - 166 | VLQPSS |
| **729.4** | 729.4 | 1+ | 16 | 28.8 | 161 - 167 | VLQPSSV |
| **844.4** | 844.4 | 1+ | 11 | 26.7 | 161 - 168 | VLQPSSVD |
| **1059.5** | 1059.5 | 1+ | 11 | 25.0 | 161 - 170 | VLQPSSVDSQ |
| **632.3** | 632.3 | 1+ | 7 | 20.4 | 163 - 168 | QPSSVD |
| **719.2** | 719.3 | 1+ | 17 | 17.0 | 164 - 170 | PSSVDSQ |
| **652.2** | 652.3 | 1+ | 10 | 20.1 | 168 - 173 | DSQTAM |
| **537.2** | 537.2 | 1+ | 9 | 20.0 | 169 - 173 | SQTAM |
| **515.2** | 515.3 | 1+ | 11 | 27.6 | 174 - 178 | VLVNA |
| **953.4** | 953.4 | 1+ | 13 | 21.8 | 189 - 196 | FKDEDTQA |
| **806.3** | 806.4 | 1+ | 18 | 16.0 | 190 - 196 | KDEDTQA |
| **593.3** | 593.3 | 1+ | 14 | 42.5 | 195 - 199 | QAMPF |
| **879.4** | 879.4 | 1+ | 13 | 35.6 | 197 - 203 | MPFRVTE |
| **1007.5** | 1007.5 | 1+ | 7 | 34.1 | 197 - 204 | MPFRVTEQ |
| **748.4** | 748.4 | 1+ | 19 | 30.1 | 198 - 203 | PFRVTE |
| **876.4** | 876.5 | 1+ | 8 | 28.6 | 198 - 204 | PFRVTEQ |
| **779.4** | 779.4 | 1+ | 13 | 26.6 | 199 - 204 | FRVTEQ |
| **815.4** | 815.4 | 1+ | 10 | 17.8 | 204 - 210 | QESKPVQ |
| **946.5** | 946.5 | 1+ | 5 | 25.5 | 204 - 211 | QESKPVQM |
| **687.3** | 687.4 | 1+ | 16 | 17.4 | 205 - 210 | ESKPVQ |
| **818.4** | 818.4 | 1+ | 15 | 25.3 | 205 - 211 | ESKPVQM |
| **949.4** | 949.4 | 1+ | 19 | 30.6 | 205 - 212 | ESKPVQMM |
| **593.3** | 593.3 | 1+ | 22 | 38.6 | 213 - 217 | YQIGL |
| **710.3** | 710.4 | 1+ | 5 | 34.5 | 218 - 223 | FRVASM |
| **565.2** | 565.3 | 1+ | 9 | 19.5 | 224 - 228 | ASEKM |
| **806.4** | 806.4 | 1+ | 19 | 28.6 | 224 - 230 | ASEKMKI |
| **1048.6** | 1048.6 | 1+ | 13 | 33.2 | 224 - 232 | ASEKMKILE |
| **761.5** | 761.5 | 1+ | 7 | 32.3 | 227 - 232 | KMKILE |
| **874.6** | 874.5 | 1+ | 14 | 41.9 | 227 - 233 | KMKILEL |
| **615.4** | 615.4 | 1+ | 16 | 39.4 | 229 - 233 | KILEL |
| **823.4** | 823.4 | 1+ | 14 | 39.6 | 233 - 240 | LPFASGTM |
| **579.3** | 579.3 | 1+ | 20 | 23.5 | 234 - 239 | PFASGT |
| **797.3** | 797.3 | 1+ | 20 | 28.4 | 234 - 241 | PFASGTMS |
| **685.3** | 685.4 | 1+ | 19 | 34.1 | 244 - 249 | VLLPDE |
| **829.3** | 829.4 | 1+ | 13 | 37.3 | 246 - 253 | LPDEVSGL |
| **722.3** | 722.4 | 1+ | 13 | 42.6 | 257 - 262 | ESIINF |
| **619.3** | 619.3 | 1+ | 18 | 21.5 | 263 - 267 | EKLTE |
| **822.4** | 822.4 | 1+ | 5 | 41.3 | 267 - 273 | EWTSSNV |
| **594.2** | 594.3 | 1+ | 8 | 19.2 | 268 - 272 | WTSSN |
| **901.5** | 901.5 | 1+ | 16 | 25.9 | 275 - 281 | EERKIKV |
| **1177.7** | 1177.7 | 1+ | 11 | 36.8 | 275 - 283 | EERKIKVYL |
| **935.6** | 935.6 | 1+ | 11 | 27.7 | 276 - 282 | ERKIKVY |
| **1048.6** | 1048.7 | 1+ | 7 | 36.4 | 276 - 283 | ERKIKVYL |
| **662.3** | 662.3 | 1+ | 13 | 26.3 | 284 - 288 | PRMKM |
| **725.3** | 725.3 | 1+ | 6 | 29.8 | 307 - 313 | FSSSANL |
| **374.9** | 748.4 | 2+ | 17 | 26.5 | 309 - 316 | SSANLSGI |
| **521.1** | 521.3 | 1+ | 12 | 20.7 | 314 - 319 | SGISSA |
| **1132.6** | 1132.6 | 1+ | 16 | 23.8 | 323 - 333 | KISQAVHAAHA |
| **605.4** | 605.3 | 1+ | 6 | 16.2 | 327 - 332 | AVHAAH |
| **676.3** | 676.4 | 1+ | 26 | 18.6 | 327 - 333 | AVHAAHA |
| **805.4** | 805.4 | 1+ | 20 | 18.4 | 327 - 334 | AVHAAHAE |
| **918.5** | 918.5 | 1+ | 46 | 27.2 | 327 - 335 | AVHAAHAEI |
| **1161.6** | 1161.6 | 1+ | 23 | 24.2 | 327 - 337 | AVHAAHAEINE |
| **605.3** | 605.3 | 1+ | 11 | 17.6 | 328 - 333 | VHAAHA |
| **632.3** | 632.3 | 1+ | 11 | 16.5 | 334 - 339 | EINEAG |
| **675.3** | 675.3 | 1+ | 11 | 15.3 | 336 - 341 | NEAGRE |
| **687.4** | 687.4 | 1+ | 7 | 21.9 | 338 - 344 | AGREVVG |
| **845.4** | 845.4 | 1+ | 22 | 22.6 | 338 - 346 | AGREVVGSA |
| **432.1** | 432.2 | 1+ | 19 | 18.1 | 342 - 346 | VVGSA |
| **632.3** | 632.3 | 1+ | 6 | 20.3 | 342 - 348 | VVGSAEA |
| **790.4** | 790.4 | 1+ | 14 | 25.1 | 346 - 354 | AEAGVDAAS |
| **550.2** | 550.2 | 1+ | 17 | 17.0 | 354 - 358 | SVSEE |
| **1131.6** | 1131.6 | 1+ | 7 | 40.5 | 358 - 366 | EFRADHPFL |
| **1002.5** | 1002.5 | 1+ | 11 | 39.9 | 359 - 366 | FRADHPFL |
| **742.4** | 742.4 | 1+ | 10 | 27.9 | 360 - 365 | RADHPF |
| **855.5** | 855.4 | 1+ | 18 | 36.4 | 360 - 366 | RADHPFL |
| **1046.5** | 1046.5 | 1+ | 21 | 32.6 | 367 - 375 | FCIKHIATN |
| **785.4** | 785.4 | 1+ | 8 | 24.5 | 368 - 374 | CIKHIAT |
| **899.5** | 899.5 | 1+ | 13 | 22.7 | 368 - 375 | CIKHIATN |
| **970.5** | 970.5 | 1+ | 12 | 24.5 | 368 - 376 | CIKHIATNA |
| **682.4** | 682.4 | 1+ | 12 | 23.4 | 369 - 374 | IKHIAT |
| **796.4** | 796.5 | 1+ | 16 | 21.3 | 369 - 375 | IKHIATN |
| **701.4** | 701.4 | 1+ | 9 | 42.2 | 372 - 378 | IATNAVL |
| **629.3** | 629.3 | 1+ | 5 | 33.6 | 379 - 383 | FFGRC |
